# Supporting patients to use online services in general practice: focused ethnographic case study

**DOI:** 10.3399/BJGP.2024.0137

**Published:** 2025-03-11

**Authors:** Jennifer Newbould, Carol Bryce, Stephanie Stockwell, Bethan Treadgold, John Campbell, Christine Marriott, Emma Pitchforth, Laura Sheard, Rachel Winder, Helen Atherton

**Affiliations:** RAND Europe, Cambridge.; Unit of Academic Primary Care, Warwick Medical School, University of Warwick, Coventry.; RAND Europe, Cambridge.; Exeter Collaboration for Academic Primary Care, University of Exeter Medical School, Faculty of Health and Life Sciences, Exeter.; Exeter Collaboration for Academic Primary Care, University of Exeter Medical School, Faculty of Health and Life Sciences, Exeter.; NIHR Applied Research Collaboration South West Peninsula Patient Engagement Group, UK.; Exeter Collaboration for Academic Primary Care, University of Exeter Medical School, Faculty of Health and Life Sciences, Exeter.; Health Sciences, University of York, York.; Exeter Collaboration for Academic Primary Care, University of Exeter Medical School, Faculty of Health and Life Sciences, Exeter.; Primary Care Research Centre, University of Southampton, Southampton.

**Keywords:** primary care, digital health, access to primary care

## Abstract

**Background:**

In England online services in general practice encompass a range of provision from ordering repeat medication to having a consultation. Some groups of individuals may find accessing and/or using such services difficult and may require ‘digital facilitation’, that is the range of processes, procedures, and personnel which seeks to support NHS patients in their uptake and use of online services.

**Aim:**

To gain insight, from the perspective of general practice staff and patients/carers, into how and why digital facilitation might lead to benefits, and the key processes involved in supporting patients to use online services.

**Design and setting:**

Eight general practices across England with varied geographical and sociodemographic characteristics were included in the study.

**Method:**

This was a focused ethnographic case study of observations and interviews (*N* = 69).

**Results:**

Typically, digital facilitation was delivered in an *ad hoc* fashion to individual patients. Online services were delivered via multiple systems each working differently and creating a need for support so that patients could access them. Younger practice staff were expected to deliver support on account of their age, despite there being no evidence of age-related training and experience. It was understood by practice staff that patients with challenging personal circumstances may require specific support to access online services.

**Conclusion:**

At present patient use of online services is supported by digital facilitation that is primarily delivered by reception staff. Supporting patients to use online services requires review of how many services are provided and what for, and consideration for the time and effort needed to support patients to use them.

## Introduction

Patient-facing online services are a growing part of arranging and delivering health care, including in general practice settings.^1^^–^3 From ordering prescriptions through to conducting the consultation, they are now a key feature of general practice in many countries including the UK.^4^

Although the use of online services in general practice is growing, uptake has been patchy. For example, use of video consultations remains low in Northern European countries, including the UK, at around 2%.^5^ However, in Denmark 25% of all consultations are delivered via email each year^6^ and in the UK 51% of all registered patients are registered to order repeat prescriptions online.^7^ These varied rates of use may reflect differing levels of digital literacy. Ten million people in the UK lack the basic skills needed to use digital services^8^ and 33% of those without online access say it is difficult to interact with NHS services.

There are some groups of patients for whom using digital services may be challenging. Individuals from minority ethnic groups, older age groups, those from lower socioeconomic groups, those with poorer health and those who live in rural settings have been shown to encounter difficulties with online approaches to accessing NHS care.^9^^–^11

NHS England have provided advice to practices in how to support patients getting online and accessing services.^12^ Alongside this, the Royal College of General Practitioners have developed a patient online toolkit to help practices provide effective online services.^13^ Despite these initiatives, levels of use of online services in general practice remain modest.

Recognising the challenges faced by many patients in using online services, the Di-Facto project^14^ is a programme of work aiming to explore the benefits and challenges of different approaches used to support online service use for patients and staff. The authors defined this support as ‘that range of processes, procedures, and personnel which seeks to support NHS patients in their uptake and use of online services’ and referred to this as ‘digital facilitation’.

Here the results are reported of one study within the wider project. A team-based focused ethnographic case study in general practices across England was conducted to gain insight, from the perspective of practice staff and patients/carers, into the potential benefits and challenges associated with different approaches to digital facilitation in general practice.

**Table:** How this fits in

Use of, and access to, online services are increasing within general practice in England. Current approaches to digital facilitation, as observed in this study, appeared to be *ad hoc* and fitted around multiple services. Reception staff were key to supporting patients to use these platforms, but training, resources, and support for such staff were not readily available. Enabling patients to have the best chance of using online services requires vision, strategy, and investment of time and money. As practices and patients increasingly use online approaches to healthcare provision, practices should be mindful of patient groups who may find accessing services online challenging and who thus require targeted help and support.

## Method

Focused ethnography, unlike traditional ethnographic approaches, enables shorter and more intensive periods of ethnography focused on a predetermined topic.^15^ This study was conducted in general practices in England, between September 2021 and July 2022. It is reported in line with standards for reporting qualitative research.^16^

It was aimed to explore, in depth, how general practice supports patients in their use of online services, to include the processes, procedures, and personnel involved.

### Sampling and recruitment

The study sought to recruit eight general practices, seeking variation in how practices reported supporting patients in using online services and by practice characteristics including setting (rural/urban), Index of Multiple Deprivation score (to ensure inclusion of practices in deprived areas), which runs from 1–10, with 1 being the most deprived. This score is available for every English general practice via National General Practice Profiles.^17^

General practices were identified via a survey within the broader Di-Facto study.^14^ The survey recruited 156 general practices in England and asked about digital facilitation services they currently offered. As well as using the survey results, practices with relevant characteristics were approached via clinical research networks or directly by the research team and invited to participate.

During fieldwork, up to six relevant staff per practice were invited to take part in a semi-structured interview. Up to six patients/carers were recruited per practice to take part in a semi-structured interview and were selected to ensure a range of characteristics across the sample in terms of age, ethnicity, and experience (or not) of being supported to use online services. The general practice sent patients/carers an invitation to participate.

Ethical approval was received from the Newcastle and North Tyneside 2 Research Ethics Committee (June 2021, reference number: 21/NE/0079) and Health Research Authority approval was also received (July 2021, IRAS number: 289425, protocol: L01886). Additionally the study received Confidentiality Advisory Group approval (21/CAG/0093.) All researchers conducting fieldwork obtained a research passport.

### Data collection and processing

Three ethnographers (the second, third, and fourth authors) were embedded in general practices during data collection, building relationships and immersing themselves in the practice environment. Data collection included non-participant observation, informal conversations, attending practice meetings, collecting relevant documentation, and conducting semi-structured interviews with staff and patients/carers. The number of hours researchers spent in each practice varied depending on the prevalence of the phenomenon of interest occurring (digital facilitation) and continued until no additional data was evident to the researcher across the data sources at each site, and disconfirming views had been obtained.

To ensure that the focus on how general practice supports patients in their use of online services was maintained across the team and multiple sites, a case study guide was created (see Supplementary Information S1). This provided a framework to ensure the researchers maintained their focus on observing any processes and procedures related to supporting patients to use online services, identifying the personnel involved. The authors have used this approach previously in team-based focused ethnography.^15^^,^^18^

Data acquisition took place in relevant parts of the surgery – for example, in reception and in administrative offices (although not in consultations). Observations and informal conversations were recorded in handwritten fieldnotes, without use of identifiers or names. Where possible, fieldnotes were made at the time of observation or discussion but if not, were written up as soon as possible afterwards. All ethnographers transferred their fieldnotes into electronic format once out of the field. Each staff member had the right to decline participation; no one exercised this option. No identifiable information about patients or carers was recorded during observations or informal conversations.

As this was a team-based ethnography, it was ensured that data was collated in a standardised format for analysis. This was achieved by using a separate document for each general practice, collating contextual information about each practice, summarised fieldnotes, listing details about who was interviewed, and noting down documentation collected. These documents were updated throughout data collection.

Separate topic guides (see Supplementary Information S2 and S3) were developed for staff and patient/carer interviews. These were informed by a review of the literature^19^ and the survey of general practices.^14^ The staff topic guide covered drivers for supporting online access in the practice, the type of support in use (resources, processes, valued outputs), the perceived success of this and challenges to implementation. The patient/carer topic guide explored use of online services outside of health, challenges to using online GP services, and participants’ experiences of digital support with the practice.

Interviews with staff were conducted within the general practice or via Microsoft Teams. For patients/carers, interviews were conducted either in person, by video, or telephone. Consent was obtained before all interviews. Interviews were audio-recorded using encrypted recorders. Patient/carer participants were given a £10 shopping voucher to thank them for their time.

Interviews were transcribed verbatim by a professional transcription service. Transcripts were then read, anonymised, and checked for accuracy by the researcher who conducted the interview.

### Research team

Researchers in the field were trained in observation techniques and were experienced qualitative researchers with varied previous experience of ethnographic approaches. Researchers who were in the field met together fortnightly during the data-collection process and met monthly with experienced researchers forming the rest of the ethnographic team. These meetings enabled discussion of the practical aspects of data collection and provided researchers with space for reflexivity. Researchers were also encouraged to diarise their reflections with their fieldnotes and share these with team members.

### Data analysis

Analysis was conducted by the authorship team using thematic analysis^20^ following four steps.

- Reading transcripts, practice summaries, and fieldnotes and developing coding frames. The practice summaries and a selection of transcripts were read by the research team including the patient and public involvement and engagement co-investigator (the sixth author).
- Developing a patient/carer, and a practice/staff coding frame (to include all fieldnotes and interview data) in two team analysis meetings. Analysis of relevant data and documentation obtained from each case study site was used to provide context.
- Gathering related sections of transcripts, fieldnotes, and documents under thematic codes; ethnographers coded data using Microsoft Word templates for each code.
- Applying thematic analysis to each line of argument in the text, looking for outliers and negative accounts; ‘one sheet of paper’ method^21^ was used to collate coded data into initial groupings. These were then used to create thematic one-page summaries. These were created by all members of the research team, before they were extensively discussed and refined to finalise themes. The summary findings were discussed within the research team and the interpretation was finalised.

## Results

Eight general practices participated, located in four different regions in England and varying in relation to size, area, deprivation score, ethnicity, and percentage of patients aged ≥65 years (Table 1).

**Table 1.** Characteristics of participating practices

**Practice ID**	**Size^a^**	**Location**	**Patients from ethnic minority, %**	**Patients ≥65 years, %**	**Index of Multiple Deprivation score^17^^,^^21^ from 1**–**10**
A	Large	Semi-rural	4.2	23.4	10
B	Small	Urban	85.7	7.3	1
C	Medium	Urban	40.0	9.4	3
D	Large	Urban	1.5	23.9	9
E	Large	Rural	1.8	8.3	5
F	Large	Rural	1.2	33.4	6
G	Large	Urban	6.4	19.6	8
H	Small	Urban	1.0	14.7	2

a *Small <6000 registered patients; medium 6001–12 000; large ≥12 000.*

Researchers spent 45–76 hours in each practice over a period of 2–6 weeks. Across participating practices, 36 practice staff, 26 patients, and seven carers were interviewed. Participating staff were from a range of roles and age groups (Table 2) and patients had a range of characteristics (Table 3).

**Table 2.** Characteristics of practice staff interviewed

**Characteristic**	**Participant, *n* (*N* = 36)**
**Gender**	
Female	23
Male	13

**Age group, years**	
18–24	3
25–34	10
35–44	7
45–54	7
55–64	4
≥65 years	1
Unknown	4

**Role in practice**	
Practice manager	8
Receptionist/admin	9
Data/IT/business manager	4
GP	8
Nurse/healthcare assistant	3
Paramedic	1
Clinical pharmacist	2
Social prescriber	1

*IT = information technology.*

**Table 3.** Characteristics of patients interviewed

**Characteristic**	**Participant, *n* (*N* = 36)^a^**
**Gender**	
Female	18
Male	15

**Age group**	
18–24	2
25–34	3
35–44	4
45–54	5
55–64	6
65–74	6
75–84	4
≥85 years	2
Undisclosed	1

**Ethnicity**	
White British	26
Asian	6
Black Caribbean	1

**Health (self-defined)** ^b^	
Long term condition(s)	13
Disability	1

**Carer**	
Yes	7
No	26

a *Three participants did not provide this information.*

b *Self-defined health reported here to indicate variation in the patient sample, reflective of typical general practice patient composition.*

Thematic analysis identified three themes that ‘set the scene’ for digital facilitation:

- COVID-19 context;
- the type of digital facilitation; and
- the perceived value and purpose of digital services.

Five themes were then presented that were relevant to digital facilitation:

- responsibility for digital facilitation;
- conflating access with online services;
- patchwork digital landscape;
- accessibility for different patient groups; and
- assumptions and stereotypes.

### COVID-19 context

Changes brought about because of the COVID-19 pandemic had an impact on the delivery of online services. The pandemic was observed to act as a catalyst for change, leading to the introduction of new online services in some practices that were not already using these:

*‘And then it, it was almost like we were just moving slowly towards, you know, some remote working and getting laptops and eConsults. And then all of a sudden, the pandemic just accelerated all of that through, through the need to manage it that way.’*

(Interview: practice A, GP partner, female)

The rapid nature of the pandemic meant that there had not been time for strategic consideration about how to introduce new online services.

### Type of digital facilitation

A range of approaches to providing digital facilitation were observed — this included support in using online platforms, information targeted at patients/carers via posters or the website, and family and friends supporting patients. Administrative staff were most often delivering digital facilitation; frequently seeking solutions to technical problems and providing *ad hoc* and ‘it will do’ support in response to patient requests:

*‘A patient came in for a face-to-face consultation as they had issues with the patient access app access. They can see their child’s (8yrs old) account on the app but not their own and need to order medication for their child. The receptionist unconfidently tried a couple of things to resolve the problem that did not work. Then the receptionist tried unlinking and re-linking the patient’s account. The patient said they could now see their account but that they (the parent) were under the proxy access of their child, rather than the child being under the parent.’*

(Fieldnote: practice H)

Examples were given of digital facilitation initiatives that were running pre-pandemic but had been paused. Examples included asking patients to bring in their devices if they needed support or holding workshops to help patients with their digital skills.

### Perceived value and purpose of digital services

In the general practices the rationale for offering patients online services in the first place was not necessarily clear. There was a lack of shared understanding among practice staff about what online services were supposed to achieve, how, and who was responsible for ensuring they were used:

*‘I’m not massively keen on policies and stuff like that. I tend to just go with strategy and in terms of being reactive to what we’ve, we get back in terms of feedback.’*

(Interview: practice A, practice manager, female)

This extended to a lack of shared vision as to what ‘good’ online services looked like and what practices were aspiring to deliver. However, patients and practice staff were united in their positivity about online repeat prescription services, and they were given as an exemplar:

*‘Male member of staff thinks prescription requests done by email are easier for the patients and the reply to say it has been done feels more personal (even though it is a template message).’*

(Fieldnote: Practice H)

Online services were difficult to deliver if patients did not want what was being offered and this was perceived by staff as the main barrier to the use of digital services:

*‘I think getting them online, like, isn’t necessarily a problem. I think it’s more whether they want to is more of a problem.’*

(Interview: practice G, receptionist, female)

Practices had not necessarily considered how to get patients to start using these services and what support was needed.

### Responsibility for digital facilitation

There was a lack of clarity over who had responsibility for digital facilitation, with practice staff and patients suggesting when asked that digital facilitation is the responsibility of ‘others’ rather than themselves. Clinical staff would routinely refer patients to administrative and reception staff to deliver digital facilitation:

*‘[...] often I have perhaps directed patients to our admin staff or our receptionists if they needed to get set up with that. […] I guess use of online services that would be more for admin or reception staff.’*

(Interview: practice H, GP [salaried], female)

General practice staff believed that patients bore some responsibility for being able to access general practice, particularly that family and friends might provide this support. In a practice with a high proportion of patients from minority ethnic groups (85.7%), there was the perception from practice staff that family were very engaged in helping the patients to use health care in general. In practice B, administrative staff often encouraged patients/carers to obtain support from family and friends:

*‘She feels that different skills are needed for using online technology, so if a person is not IT* [information technology] *literate the administrative staff will ask the patient to get a family member or grandchildren involved to help them.’*

(Fieldnote: Practice B)

In practice D, which was large and had a very low number of patients from minority ethnic groups (1.5%), a patient remarked that the responsibility for supporting patients to use online services did not belong to the practice:

*‘It’s difficult, isn’t it? I, I don’t see the role of the practice to be educating us in, in how to adopt technology. I think it’s just too much. I think the remit of the NHS is so wide.’*

(Interview: Practice D, patient, male, aged 45–54 years)

Although views were not consistent about who should be responsible, the reality was that there was no formal designation of responsibility, and reception staff in practices were conducting the majority of the digital facilitation where it was needed.

Providing patients with adequate support was thought to require lots of input, planning, time, funding, and training from somewhere, but tended to be thought of as something that should be provided from outside the general practice:

*‘Then it has to come from the NHS and government as well at the same time. Well, what provision have you put into place? Or what funding have you put into place that we can give time to patients.’*

(Interview: practice C, GP partner, male)

Digital facilitation was largely occurring in an *ad hoc* way, without clear lines of responsibility, without strategy, and without consistent health service investment. There was deemed to be a lack of national guidance or support.

### Conflating access with online services

Patients viewed online services as one of the routes to access the general practice. Any kind of remote access including the telephone, was referred to as part of online services. When asked about online services, a patient referred to a telephone service:

*‘I call* [automated telephone line ] *for the prescription medicine order online.’*

(Interview, practice B, patient, female, aged 25–34 years)

This reflected the complexity of services offered, with some online services leading to a telephone call back, and online platforms being offered in lieu of telephone consultations.

Access to speaking to, or seeing, a health professional through any medium was of central importance to patients:

*‘I wouldn’t even start to try and book an online appointment with my GP. Because if I did that, I would probably be offered something in 4 to 5 weeks’ time. And my only choice, if I want to see a doctor, urgent or not, is to phone up at 8.30 on the morning and fight with everybody else for an appointment on the phone, for a that-day appointment.’*

(Interview: practice D, patient, female, aged 35–44 years)

The priority of patients was to achieve access as quickly as possible in whichever way was possible. The onus was on patients to understand and use online services if they wished to have this additional route of access available to them.

### Patchwork digital landscape

Within the range of online services there was great variation. All general practices offered patients access to more than one online service system and these varied greatly in purpose and usability, for example, one may be for consultations, another for ordering repeat prescriptions and making appointments. Practice staff routinely had to work across multiple online systems that were not necessarily compatible with each other. Sometimes general practices used the services interchangeably:

*‘[...] so if you can’t manage the text message, I’ve talked people through how to go online and do an eConsult and upload the photographs that way.’*

(Interview: practice A, GP partner, female)

Initial registration processes to use online services could be complex for patients and were often associated with a need for digital facilitation:

*‘Because I kept doing it and it wasn’t accepting it and it wasn’t registering. And it was frustrating because I couldn’t do. And I was there forever, and my battery was dying, and I’m thinking, “I need to get this done but I can’t do it”.’*

(Interview: practice B, patient, female, aged 45–54 years)

The need for digital facilitation was related to how user friendly the online services were, with some online services more complex to access and navigate, and this was clearly acknowledged by general practice staff and patients alike:

*‘I do think the patients need educating on it, and I do think the* [online consultation system] *itself is, is clunky, because I’ve looked at it from a patient perspective on our* [online consultation system], *and it, you just get caught in a minefield of information.’*

(Interview: practice G, quality and performance manager, female)

Some of the online services were regarded as user friendly, did not present problems, and appeared to be a good fit for patients:

*‘As long as I follow the instructions they’re, they’re quite easy.’*

(Interview: practice C, patient, male, aged 65–74 years)

Interoperability issues between systems could easily terminate the patient digital journey. Even for patients who are very digitally engaged, the use of multiple systems appeared to have the potential to cause problems with patient access to online services.

The NHS App is a nationwide service operated centrally by the NHS and linked to all NHS services that a patient uses. This means that general practice can only provide limited support for those using it as it is external to the practice:

*‘So, it’s a bit difficult because the NHS App is a third-party app there is obviously only so much we can do our end.’*

(Interview: practice G, receptionist, female)

As the NHS App is a main route for patients to access online services, the fact that general practice had no control over individual patient access to it was a considerable issue.

### Accessibility for different patient groups

The challenges faced by some patients were greater when it came to online services. Examples identified in the case study practices included internet-related circumstances, such as patients in rural areas with poor internet connections and those with no internet connection at all. There were patients who already faced communication barriers, particularly those who do not speak English:

*‘… with language barriers as well, because they don’t really understand what’s being asked of them and what they need to do.’*

(Interview: practice A, receptionist, female)

Individual circumstance could influence a need for support, especially for those in challenging circumstances such as people who are homeless, patients with learning disabilities, those with sensory impairment, and those in lower socioeconomic groups:

*‘They’ve got to watch their data, you know, and if they’re not on Wi-Fi they can use a lot of data, especially homeless people, you know, who haven’t got access to Wi-Fi perhaps, and they, they all generally afford about a tenner a month for the phone. But the data for that online, they might not do it* [the cost of data for accessing online services would be more than ten pounds].’

(Interview: practice H, carer, male, aged 55–64 years)

General practices themselves applied an individual approach to the circumstances of patients. They described making adaptations for patients with different needs in the hope that this was enough to facilitate access to online services, but without knowing if it did.

Age was perceived to have an impact on staff and patient ability to use online services, particularly in relation to their experience of and confidence with online services. For older patients, using online services was about more than ability, but also about trusting online services when dealing with personal matters:

*‘For banking, for ordering prescriptions, but I’m beginning to … oh, also filing my tax return … But I’m beginning to use it less and less … Security issues, basically, and the fact that people like Google are just so powerful and so invasive nowadays.’*

(Interview: practice F, patient, male, aged 65–74 years)

General practice staff shared the vision that online services should work well for every patient, regardless of their experiences and challenges, but did not have the means to put this into practice. Planning for digital facilitation was difficult when it was not clear who was most in need and least able to navigate online services, nor how this could be best addressed.

### Assumptions and stereotypes

The complexity of accessibility for different patient groups was compounded by the impact of assumptions and stereotypes. Embedded attitudes, from both patients and staff about who needs support to use online services were observed. Assumptions were made about who would or would not be able to use online services.

Age was a key example of this and participants themselves raised this. For example, it was perceived that younger patients would be more able to use online services:

*‘The youngsters, once they get, the younger people, once they tend to get up on it,* [online] *there’s no real issues.’*

(Interview: practice G, practice manager, male)

Older age was often perceived as equating to poorer online knowledge, use, and confidence and although this could be true, it was not necessarily always the case. This perception was evident among both patients and carers, as well as among staff. This had an impact on who was offered support to use online services, and how. In the following example the receptionist assumes that older people do not want to use online services:

*‘Yeah. It’s getting better, and obviously it depends on the demographics of the patient, because, you know, obviously the elderly and, and ones that aren’t so computer-literate, you know, can’t do it or aren’t interested in doing it.’*

(Interview: practice D, senior receptionist, female)

It was observed that an assumption was being made that those unable to use digital services would have some form of ‘proxy access’ either in the form of a family helper or carer who can support their online access.

Both patients and practice staff would often categorise themselves according to their technical abilities and this reflected societal perceptions about who does or does not use online services:

*‘Yeah, I, I would say, so. I mean, we use it constantly, so we use Teams at work, Outlook, as well sort of Excel. I’m an, an* [job title] *so I use* [name of online system] *systems and things like that at work as well. So, yeah, I guess compared to a lot of people who might not use the computer much at all, then, yeah, I’m reasonably tech savvy.’*

(Interview: practice D, patient, female, aged 45–54 years)

Viewpoints about age extended to views about who should be delivering support, with assumptions made by general practice staff that staff of younger age are more capable of delivering digital facilitation. Confidence, rather than competence, was viewed as important in supporting patients to use online services.

## Discussion

### Summary

Digital facilitation in general practice was focused on helping the individual patient who presented for support. The need for digital facilitation was influenced by the type and range of online services available to patients. Some online services were easier to use and more welcome than others, with online repeat prescription ordering an example of a service that was well used. A clear line of responsibility for providing digital facilitation was lacking and consequently, reception staff were providing it. This tended to be in an individual encounter where patients would be focused on achieving access to a doctor or a particular service, which may or may not necessitate the use of online services. Adaptations were made for patients who were in challenging circumstances, but the means to do this in an organised fashion were lacking. It was observed that assumptions were made about the impact of age on the ability to use online services, with older people deemed less likely to use them, and younger people more likely to be confident in using them.

### Strengths and limitations

This in-depth ethnographic study examined eight general practices, with varied geographical locations and sociodemographic characteristics. Despite the variation in types of practices, the findings were consistent across practices. Consultations were not observed as the majority of digital facilitation was known to occur outside the consultation.^14^ This meant any digital facilitation happening during consultations was not observed, although this was asked about via the semi-structured interviews. The fieldwork was conducted during a challenging time given COVID-19 restrictions, with fewer patients and carers attending the practice and waiting rooms quiet. More telephone communications with patients were observed rather than in-person exchanges.

### Comparison with existing literature

It was found that the value and purpose of the online services offered in general practice was not always known or understood. Research examining the implementation of digital technologies in a healthcare setting found that new technologies were most accepted when there was a positive impact on relationships between patients and staff, when they fit with the organisation’s goals, and match well with the skill sets of staff.^22^ The lack of understanding of the value and purpose of online services influenced how they worked, and in turn whether staff were confident in supporting patients to use them.

The NHS App has been promoted to patients in England, with levels of registrations for the app increasing.^23^ However, general practices do not retain any control over the technical elements of using it. This limits the extent to which digital facilitation can be provided. Research has shown that post-registration use of the facilities offered by the app remains proportionally low relative to the numbers registering.^24^ This fits with our observations about the challenges faced by patients in trying to use the app, and by general practice in supporting patients to use it.

Morley and Floridi have previously described ‘techno-utopia’, where digital health tools enable and support the doctor–patient relationship.^25^ The reality, at least as far as this research is concerned, is quite different, with coordination of digital facilitation efforts not evident, a lack of training and resource opportunities, and potential disenfranchisement of patients in respect of important online healthcare provision and services.

### Implications for research and practice

For the NHS to reach its ambition of greater use of online approaches in primary care^26^ there should be a more considered and coordinated approach to supporting patients. Successful digital facilitation will require funding, infrastructure, and staff training that is not currently consistently available. There should be acknowledgement from those at policy and commissioner level that merely procuring a service does not equate with successful use of that service by patients.

To reduce the need for digital facilitation in the first place, online services could be specifically designed around the needs of patients/carers and tailored to individuals’ personal needs and requirements. Practices may find benefits, for both staff and patients/carers, in avoiding the use of multiple online platforms. Practices should also consider the usability of online approaches, for staff and patients/carers, when looking to procure new systems, although this can be difficult at the onset of use, and it may take time to understand usability.

As practices use more online approaches, they will need to be adaptable to ensure inclusion of patient groups who may find accessing services online to be a challenge and may require targeted help and support – such individuals and groups of patients may be considered ‘digitally vulnerable’. Unconscious bias training may be one way to counter assumptions about the abilities of staff or patients/carers based on characteristics such as age, something which has been used successfully within the NHS^27^ and in other organisations and industries.

Further research is needed to understand what forms of digital facilitation may be most effective, allowing a move beyond *ad hoc* provision. This may take the form of training and supportive interventions for general practice staff. Understanding and monitoring the impact of poor access to online services is crucial to avoid further inequalities.

In conclusion, rather than being an organised activity, at present, patient use of online services is supported by digital facilitation that is primarily delivered in an *ad hoc* manner by reception staff. Supporting patients to use online services requires a review of how many services are provided and what for, and consideration given to the time and effort needed to support patients as they navigate these services.
