# Supplementary material for: Supporting patients to use online services in general practice: focused ethnographic case study
Source: Br J Gen Pract. 2025 Mar 11;75(755):e382–9. doi: 10.3399/BJGP.2024.0137 (PMC11920896; doi:10.3399/BJGP.2024.0137)
Supplement: Supplementary file 1 [file BJGP.2024.0137_suppl.pdf]

Supplementary information 1

Objective of the case studies

- To explore in-depth the use of digital facilitation in general practices. Through non-participant observation, semi-structured interviews, and secondary analysis of documentation.
- Build a detailed picture to explore what approaches practices have taken to digital facilitation, why facilitation was introduced, how they have introduced and/or implemented their approaches, what has made this possible, and what the benefits and challenges have been from the perspective of staff and patients/carers.
- Changes in the provision of primary care as a result of COVID-19 have been extensive. The case studies will focus on the models of facilitation in place at the time of the research but will explore any changes made in the light of COVID-19.

We are interested in the following groups:

Staff: GPs, nurses, nurse practitioners, reception staff, practice manager, administrative staff and any other staff involved in digital facilitation.

Patients and carers: All patients aged 18 years and over. We are particularly interested in patients who have been targeted by or participated in efforts to increase uptake of online services.

Data collection will include:

- Non-participant observation to understand how digital facilitation occurs within the practice, with whom, and in what ways.
- Attendance at practice meetings.
- Collection of relevant data and documentation.
- Interviews

Findings from the literature review in WP1 have been used to develop a typology of digital facilitation. This typology will be a useful reference for the ethnographers in identifying types of digital facilitation that the practice staff and patients are interacting with.

**Typology of digital facilitation**		**Definition**
Digital facilitation aimed at patients	Promotions	Broad category of digital facilitation that captures ways of raising awareness of and knowledge about digital services, endorsements of specific digital services to patients, and methods of encouraging patients to use them.
	Training and education	Education or training to help patients acquire technical skills to use digital services or to help patients understand what features of a digital service can be most helpful to them.
	Guidance and support	Ongoing help in using digital services provided by clinicians or other primary care staff to patients.
Digital facilitation aimed at primary care staff		Interventions aimed at primary care staff typically aim to increase staff's knowledge of digital services so that they can better support patients in their use of the services, or to increase their trust in services in order to increase the likelihood of staff promoting the service to patients.

Findings from the survey:

<u>Types of DF</u>

Passive – display, leaflet, text message and/or emails, practice website, social media.

Active – ad hoc, practice champion, workshops or events, provision of tablets/computers.

Groups practices say they target in order of % reported:

1. Older adults
2. Patients with caring responsibilities or patient carers
3. Socially isolated individuals
4. People with limited computer skills
5. Non-English speakers /English as a second language
6. People with limited/no internet access
7. BAME communities

In the following statements these were unilaterally responded to with strongly agree/agree in the survey (more than 50% of responses):

- Some patients are unlikely to use online primary care services no matter how much we support them.
- Some patient groups (e.g. older or more deprived patients) require more support in using online primary care services than others.
- We hope to increase uptake of online primary care services by supporting our patients to use them.
- We have managed to increase uptake of online primary care services by supporting our patients to use them.
- Increased patient uptake of online primary care services leads to operational efficiencies for the practice
- Online primary care services are complementary to traditional forms of access.
- Online primary care services will ultimately replace traditional forms of access.

Reception Staff * likely to be heavily involved in DF

Tasks	*Per protocol approach*	Factors to explore
• **Observation**<br>• **Interview**<br>• **Informal conversations**	• *What facilitation takes place*<br>• *What devices are used to deliver facilitation (laptop/phone/tablet)*<br>• *How do barriers to facilitation present*<br>• *How are the barriers to facilitation overcome or not*	▪ When is digital facilitation mentioned: include context<br>▪ Which type of facilitation is observed: promotion; training and education; guidance and support<br>▪ Is it passive or active or both?<br>▪ How is it decided which type of facilitation to offer?<br>▪ Do they signpost patients/carers to others in the practice or externally for help with digital services?<br>▪ How do they feel about their role in digital facilitation (including how well equipped they feel to perform this role)?<br>▪ What is their role in encouraging

		patients/carers to use digital services?<br>▪ How do they feel about this role as part of their job?<br>▪ Do they derive satisfaction from engaging in digital facilitation<br>▪ What were their perceived training needs? How have they been addressed in the past and now<br>▪ What types of patients are likely to need digital facilitation?
• **Create structured summary profiles (to be populated by asking relevant staff these specific questions)**	• *What types of digital services are provided by the practice*<br>• *What types of facilitation are routinely used*<br>• *How are patients signposted to the facilitation types and by whom*	▪ Using the typology which models of facilitation are in use?<br>▪ Are they passive or active or both?<br>▪ How long have they been in use?<br>▪ Have there been changes since the start of the COVID-19 pandemic?<br>▪ What training have staff been given on digital facilitation?<br>▪ How does the whole practice support the use of digital facilitation – whose responsibility?

Practice Managers

Tasks	*Per protocol approach*	*Factors to explore*
• **Interview**<br>• **Observation**<br>• **Informal conversation**	• *Types of digital services offered and when.*<br>• *Types of facilitation.*<br>• *Responsibility for facilitation strategy*<br>• *Strategy evaluation and resulting changes.*<br>• *Targeting of different groups in the practice population.*<br>• *Perceived benefits of new services.*<br>• *Barriers to use.*<br>• *Impact of facilitation efforts.*<br>• *Changes seen since COVID-19 pandemic*	▪ Who made decisions on the introduction of digital services?<br>▪ What were the drivers that led to the introduction of these services?<br>▪ What are the differences in uptake between patient groups in practice?<br>▪ What types of patients are likely to need digital facilitation?<br>▪ Do the practice actively work with certain patient groups to improve uptake? What are their criteria for doing so i.e.do they target those where uptake should be easier or those they know are falling behind in terms of uptake?<br>▪ How are different types of digital facilitation applied?<br>▪ Who makes decisions on facilitation methods?<br>▪ Is digital facilitation seen as a whole staff issue or some members of staff more responsible?<br>▪ Does facilitation involve anyone beyond practice staff? Do they consider what other practices do?

		▪ How are digital facilitation strategies discussed at practice meetings?<br>▪ What are the perceived benefits of different types of digital facilitation and for whom<br>▪ What were managers' perceived training needs? How have they been addressed in the past and now?<br>▪ Is it the responsibility of the practice or the wider NHS/CCG to ensure that patients receive digital facilitation?
• **Create structured summary profiles**	• *What types of digital services are provided by the practice*<br>• *What types of facilitation are routinely used*<br>• *How are patients signposted to the facilitation types and by whom*	▪ What types of digital facilitation used most actively?<br>▪ Are they passive or active or both?<br>▪ How has this changed over time?<br>▪ How have staff members been trained on facilitation?<br>▪ What changes have been implemented since COVID-19 pandemic?
• **Review practice meeting minutes and other documentation on online service introduction and digital facilitation**	• *Review **notes** and **minutes** from practice meetings where online services or digital facilitation is discussed.*<br>• *Review policies for the introduction and use of online services and digital facilitation strategies.*<br>• *Look for any templates, protocols or scripts used to*	

	automate patient engagement	

GPs

Tasks	Per protocol approach	Factors to explore
• **Interview**<br>• **Observation**<br>• **Informal conversation**	• *Types of online services they* encourage *patients to use*<br>• *Clinician's perspectives on the importance of using online services and how digital facilitation aids this.*<br>• *Benefits of the use of online services for different patient groups*<br>• *Benefits to staff of more patients using online services*<br>• *Facilitators to using different types of digital facilitation*<br>• *Barriers to using the different types of digital facilitation*<br>• *Assumptions about who would use services or benefit from digital facilitation*<br>• *Importance of outcomes from digital facilitation.*<br>• *Characteristics of GPs that might affect their promotion of online services or digital facilitation.*<br>• *Explore how GPs record their signposting to online services and digital facilitation.*	▪ Which online services are promoted to which patients and how?<br>▪ Which types of digital facilitation are suggested by the GP to different patient groups?<br>▪ Are they passive or active or both?<br>▪ What types of patients are likely to need digital facilitation?<br>▪ Do GPs feel invested in helping their patients use online services? If so, which do they help with the most?<br>▪ What are the barriers and facilitators to successful digital facilitation?<br>▪ Do GPs feel it is part of their job to facilitate their use or signpost to those who can facilitate their use?<br>▪ How does the use of online services impact on their relationship with their patients? (look for facilitators and barriers)

		▪ What outcomes of digital facilitation do they feel are important? To which groups?<br>▪ Which online services do they think have the greatest value to different patient groups?<br>▪ Have their views changed since the pandemic?<br>▪ What were their perceived training needs? How have they been addressed in the past and now?<br>▪ Is it the responsibility of the practice or the wider NHS/CCG to ensure that patients receive digital facilitation?

Nurses

Tasks	Per protocol approach	Factors to explore
• **Interview**<br>• **Observation**<br>• **Informal conversation**	• *Types of online services they encourage patients to use.*<br>• *Models of facilitation used to help patients use the services.*<br>• *Facilitators to engaging with digital facilitation.*<br>• *Barriers to engaging with digital facilitation.*<br>• *Nurse perception of digital facilitation and online services.*<br>• *Nurse perception of appropriate patient groups to*	▪ How do they feel about promoting online services?<br>▪ What types of digital facilitation do they engage with and with whom?<br>▪ Are they passive or active or both?<br>▪ What are the facilitators and barrier to this engagement?<br>▪ What are their assumptions about who would benefit most from digital facilitation?

	engage with digital facilitation.<br>• *Assumptions about who would benefit from digital facilitation.*<br>• *Facilitation tried and success of different types of facilitation.*<br>• *Importance of outcome of digital facilitation*<br>• *Impact on workload*<br>• *Explore views on responsibility for digital facilitation*<br>• *Impact on patient clinician relationship*<br>• *Explore impact of COVID-19 pandemic.*	▪ What has been successful and less successful in relation to digital facilitation models?<br>▪ How does digital facilitation and online service use impact on their relationship with their patients?<br>▪ What outcomes of digital facilitation do they feel are important and why?<br>▪ What are online services useful for in their daily practice?<br>▪ Do they feel part of the team decision making on digital facilitation?<br>▪ Who do they consider responsible for digital facilitation?<br>▪ Which digital services do they consider have the greatest value to patients?<br>▪ Do they feel their approach differs to other colleagues? Look at clinical colleagues and non-clinical colleagues.<br>▪ What is the dynamic between nurses and GPs in relation to digital facilitation?<br>▪ What were their perceived training needs? How have they been addressed in the past and now?

Administrative staff * likely to be heavily involved in DF

Tasks	Per protocol approach	Factors to explore
• **Interview**<br>• **Observation**<br>• **Informal conversations**	• *Involvement in digital facilitation*<br>• *Mechanisms by which they are approached by patients for help with online services.*<br>• *Extent to which they feel digital facilitation is part of their role*	▪ How do they deal with patient requests for online services?<br>▪ Are they involved in any digital facilitation?<br>▪ What types of patients are likely to need digital facilitation?<br>▪ Do they see digital facilitation as part of their role?<br>▪ To what extent do they understand the purpose and different types of digital facilitation?<br>▪ How engaged are they with the practice strategy on online service use and digital facilitation to enable this?<br>▪ Has the COVID-19 pandemic impacted their work in relation to digital facilitation?<br>▪ What is the dynamic between admin staff and clinical staff in relation to digital facilitation?<br>▪ What were their perceived training needs? How have they been addressed in the past and now?

Social prescribers

Tasks	Per protocol approach	Factors to explore
• **Interview**<br>• **Observation**<br>• **Informal conversations**	• *Involvement in digital facilitation*<br>• *Mechanisms by which they are approached by patients for help with online services.*<br>• *Mechanisms by which patients are signposted to them for digital facilitation.*<br>• *Extent to which they feel digital facilitation is part of their role*<br>• *Assumptions about who would benefit from digital facilitation.*<br>• *Facilitation tried and success of different types of facilitation.*<br>• *Importance of outcome of digital facilitation to patients and the practice*<br>• *Explore impact of COVID-19 pandemic.*	▪ How do they deal with patient requests for online services?<br>▪ Are they involved in any digital facilitation?<br>▪ What types of patients are likely to need digital facilitation?<br>▪ How does digital facilitation fit with other roles in the practice?<br>▪ Do they see digital facilitation as part of their role?<br>▪ To what extent do they understand the purpose and different types of digital facilitation?<br>▪ How engaged are they with the practice strategy on online service use and digital facilitation to enable this?<br>▪ What is the dynamic between different members of staff and digital facilitation?<br>▪ Has the COVID-19 pandemic impacted their work in relation to digital facilitation?<br>▪ What were their perceived training needs? How have they been addressed in the past and now?

Practice champions

Tasks	Per protocol approach	Factors to explore
• **Interview**<br>• **Observation**<br>• **Informal conversations**	• *Involvement in digital facilitation*<br>• *Mechanisms by which they approached patients or patient groups to help with online services.*<br>• *Mechanisms by which patients are signposted to them*<br>• *Assumptions about who would benefit from digital facilitation.*<br>• *Facilitation tried and success of different types of facilitation.*<br>• *Importance of outcome of digital facilitation to patients and the practice*<br>• *Explore impact of COVID-19 pandemic.*	▪ What types of digital facilitation do they engage with and with whom?<br>▪ Are they passive or active or both?<br>▪ What are the facilitators and barrier to this engagement?<br>▪ What types of patients are likely to need digital facilitation?<br>▪ What are their assumptions about who would benefit most from digital facilitation?<br>▪ What has been successful and less successful in relation to digital facilitation models?<br>▪ How does this impact on their relationship with patients?<br>▪ Do they feel the outcome of digital facilitation is important and why?<br>▪ How do they deal with patient requests for online services?<br>▪ How does digital facilitation fit with other roles in the practice?

		▪ To what extent do they understand the purpose and different types of digital facilitation?<br>▪ How engaged are they with the practice strategy on online service use and digital facilitation to enable this?<br>▪ What is the dynamic between different members of staff and digital facilitation?<br>▪ Do they feel part of the decision-making team on digital facilitation?<br>▪ Who else in the practice team do they consider has any responsibility for digital facilitation?<br>▪ Which digital services do they consider have the greatest value to patients?<br>▪ Has the COVID-19 pandemic impacted their work in relation to digital facilitation?<br>▪ What were their perceived training needs? How have they been addressed in the past and now?

Patients/Carers

**Tasks**	**Per protocol approach**	**Factors to explore**
• **Interview**<br>• **Observation**	• How do patients engage with different models of digital facilitation.<br>• Discuss the barriers and facilitators to using online services.<br>• Explore the perceived advantages and benefits to them of being able to access services online.<br>• Understanding about changes brought in over the past few years and how has this impacted their use of the general practice.	▪ How much are patients aware of the different online services and which are used most frequently?(This should be asked for contextual information not as focus of discussion.)<br>▪ Did they experience any difficulties in accessing online services?<br>▪ If so, how did they go about getting help? Did anyone do it with them – family member or in practice?<br>▪ Who in the practice helped them get online?<br>▪ In what form was that help?<br>▪ Were they targeted for help although they had not asked for it because of a particular characteristic?<br>▪ Looking at all the models of digital facilitation used in the practice which were the patient aware of and which had they used?<br>▪ Which models of facilitation were most useful and in what circumstances?<br>▪ Now that you can access online services, what are the advantages of using online services? How did digital facilitation help with this?<br>▪ Explore feelings of trust in the person at the practice that signposted both the online service and digital facilitation?

		▪ Look for issues of privacy and confidentiality and explore what they mean by these things.<br>▪ Assess whether the patient was aware that they received facilitation (use appropriate terminology).<br>▪ Where they have not used digital facilitation and/or online services, why is that?

Vulnerable groups

**Tasks**	**Per protocol approach**	**Factors to explore**
• **Interview**<br>• **Observation**	• How do patients engage with different models of digital facilitation.<br>• Discuss the barriers and facilitators to using online services.<br>• Explore the perceived advantages and benefits to them of being able to access services online.<br>• Understanding about changes brought in over the past few years and how has this impacted their use of the general practice.	▪ How much are patients aware of the different online services and which are used most frequently? (This should be asked for contextual information not as focus of discussion.)<br>▪ Did they experience any difficulties in accessing online services?<br>▪ Do they recognise themselves as being someone who might benefit from digital facilitation or face particular barriers to online services?<br>▪ If so how did they go about getting help? Is this from family/friends other sources?<br>▪ Who in the practice helped them get online?<br>▪ In what form was that help?<br>▪ Were they targeted for help although they had not asked

		for it because of a particular characteristic?<br>▪ How do they feel about being sought out for help?<br>▪ Looking at all the models of digital facilitation used in the practice which were the patient aware of and which had they used?<br>▪ Which models of facilitation were most useful and in what circumstances?<br>▪ Now that you can access online services, what are the advantages of using online services? How did digital facilitation help with this?<br>▪ Explore feelings of trust in the person at the practice that signposted both the online service and digital facilitation?<br>▪ Look for issues of privacy and confidentiality and explore what they mean by these things.<br>▪ Assess whether the patient was aware that they received facilitation (use appropriate terminology).<br>▪ Where they have not used digital facilitation and/or online services, why is that?

Supplementary information 2

Topic Guide: Staff Interview

Study title: Di-Facto: Digital Facilitation in Primary Care

Introduction

Our current research project looks at digital facilitation, how GP practices support patients and carers to access online GP services. This might include helping a patient to access services to order medication online, helping a patient to message a health professional, or showing a patient how to upload a photo of their skin condition to a health professional, to give just a few examples. As researchers we are not for or against any particular way of supporting patients to access services online, but we would like to understand more about professionals' views and experiences.

Does that sound clear to you? Is there anything you would like to ask before we start the interview?

[TAKE CONSENT]

1. Can you tell me your role at the practice?

   If GP – are they a partner? If nurse – HCA, practice nurse, nurse practitioner?

   Admin staff – what is their role – do they work on reception at all?

   How long have they worked at the practice?

2. Can you tell me a bit about the practice and the population that you serve? What does a typical day in the practice look like for you?

3. Can you tell me about the online services the practice uses? Which are you involved in?

   Medication ordering
   Booking appointments
   Obtaining test results
   Messaging the GP/using online consultation
   Other

4. Can you tell me about your views and experience of getting patients or carers to use online services at your practice?

   Do you know what proportion of patients at the surgery access services online?
   Do you encourage patients to access services online? How you do you feel about doing this? For which sorts of services; ordering medication, booking an appointment, messaging a health professional, uploading a photo to a health professional.

5. What are the reasons the practice encourages patients to access online GP services?

    Patient demand
    COVID-19 pandemic
    Policy initiatives which have encouraged practices to do so
    GP contract requirements to do so
    Does greater use of online services impact on how you can run the practice – for example freeing up administrative time, avoiding lots of demand first thing in the morning?
    Are there any barriers to doing this?

6. Does the practice have a specific approach to helping patients and carers to access online GP services?

    If yes can you tell us a bit more about that? What does that look like?
    If not, is this something done by all professionals in the practice or some?
    Where does it sit in list of priorities?

7. Can you tell us something about the patients you support to use online GP services?

    What sorts of patients do they tend to be?
    What factors impact on online access to GP services – disability, age, ethnicity, English as not the first language, access to devices, adequate internet access?
    Do you feel more confident supporting certain groups over others?
    Are the patients you provide support to different from the patients you encourage to use online services?

8. I want to ask about some of the ways in which the surgery has helped to get patients and carers to access GP services online. What help has the practice given patients?

    Who has provided that support – staff? PPG members? Other?
    How successful have you found efforts to get patients to use online GP services have been? What has helped in getting patients to use online services?

9. What challenges have you encountered trying to get more patients online?

    Time/ resource?
    Do practice staff have the skills/resources to deliver this?
    COVID-19 restrictions or impacts on practice resources?
    Lack of national guidance or support in how to do it?
    Patients are not willing to receive such support?

10. Is there anything else we have not discussed which you'd like us to talk about?

Across the whole study we are gathering data from individuals in three areas of England and when we are looking at the data overall it is really useful to have some information about those who have taken part in the interviews. I have three questions I would like to ask you about yourself and it is entirely up to you if you wish to answer them or not.

What is your gender? What age or age group are you? What ethnicity are you?

Thank you very much for your time today.

UNIVERSITY OF EXETER

RAND EUROPE

WARWICK
THE UNIVERSITY OF WARWICK

FUNDED BY
NIHR | National Institute for Health Research

This study is funded by the National Institute for Health Research (NIHR) [Health Services and Delivery Research Programme 128268]. The views expressed are those of the author(s) and not necessarily those of the NIHR or the Department of Health and Social Care.

Supplementary information 3

Topic Guide: Patient/ Carer Interviews

Study title: Di-Facto: Digital Facilitation in Primary Care

Introduction

Our current research project looks at how GP surgeries support patients and carers to access online GP services such as ordering a medication, making an appointment or messaging the GP. We are really interested in finding out more about your experiences of support from the practice in accessing online services. As researchers we are not for or against any particular way of accessing services online, but we would like to understand more about what people really think.

Does that sound clear to you? Is there anything you would like to ask me at this stage?

[TAKE CONSENT]

1. I'd like to start by just asking generally about how you use online services currently. Do you use online services for things like shopping, banking or chatting with family or friends?

    How do you feel about using online services?

2. Thinking now about your GP surgery, how long you have been a patient at the XX (name of practice) surgery?

3. How often do you tend to use the GP surgery?

4. Do you know which online services your GP surgery offers to patients?

    After leaving the interviewee to think: Prompt (depending on what you know is available at the surgery)

    Ordering medicines online
    Making appointments online
    Having a consultation online
    Messaging a doctor or other professional
    Uploading photos

5. Which of the online services available at your practices have you used?

    Ordering medicines online
    Making appointments online
    Having a consultation online
    Messaging a doctor or other professional
    Uploading photos

6. How often do you use these services?

7. What are your main reasons for using online services from your GP surgery?

Were you encouraged by someone else, if so who?
How easy is it to navigate things from the surgery not online e.g. to get through on the telephone, or to get a face to face appointment?
Has COVID-19 changed things, not being able to visit the practice in person?

8. How do you feel about going online to access services from your GP?
   Have you done so? What did you do?
   How did you find it?
   What device did you use?
   Does anyone help you when you do this?

9. Are there things that affect whether and how you access online services?
   Personal factors – e.g. sight difficulties
   Technology factors – e.g. lack of device, lack of internet access, problems with internet access
   The process of registering for online services

10. Have you had any help from the practice in getting online? Can you describe the help that you have received?

    What was nature of the help?
    Who was it with? – member of GP staff, PPG member etc.
    How useful was the help that you had?
    Have you since accessed services for your GP surgery online since this help? If yes- how did you find this?

11. Do you feel that more help from your GP surgery would be useful in accessing online GP services?

If so, can you describe what sort of help would be useful from your practice to support your use online services?

If yes – what sort of help would be useful? From whom, and in what ways?

12. Is there anything else about online services which we have not talked about, but you would like to mention?

For the whole study we are gathering data from individuals across three areas of England and when we are looking at the data overall it is really useful to have some information about those who have taken part in the interviews. I have three questions I would like to ask you about yourself and it is entirely up to you if you wish to answer them or not.

What is your gender? What is your age or age group? What is your ethnic group? (Alternative what is your ethnicity?)

Thank you very much for your time today.

FUNDED BY NIHR | National Institute for Health Research

This study is funded by the National Institute for Health Research (NIHR) [Health Services and Delivery Research Programme 128268]. The views expressed are those of the author(s) and not necessarily those of the NIHR or the Department of Health and Social Care.
